# TRustDB: A comprehensive bioinformatics resource for understanding the complete Wheat—Stem rust host–pathogen interactome

**DOI:** 10.1093/database/baac068

**Published:** 2022-11-17

**Authors:** Raghav Kataria, Rakesh Kaundal

**Affiliations:** Department of Plants, Soils, and Climate, College of Agriculture and Applied Sciences, Utah State University, Logan, UT 84322, USA; Department of Plants, Soils, and Climate, College of Agriculture and Applied Sciences, Utah State University, Logan, UT 84322, USA; Bioinformatics Facility, Center for Integrated BioSystems, College of Agriculture and Applied Sciences, Utah State University, Logan, UT 84322, USA; Department of Computer Science, College of Science, Utah State University, Logan, UT 84322, USA

## Abstract

The increasing infectious diseases in wheat immensely reduce crop yield and quality, thus affecting global wheat production. The evolution in phytopathogens hinders the understanding of the disease infection mechanisms. TRustDB is an open-access, comprehensive database that is specifically focused on the disease stem rust (also known as black rust) in *Triticum aestivum*, which is caused by the fungal pathogen *Puccinia graminis* (*Pgt*), strains ‘Ug99’ and ‘21-0’. The database aims at a broader focus of providing the researchers with comprehensive tools to predict the protein–protein interactions and avail the functional annotations of the proteins involved in the interactions that cause the disease. The network of the predicted interactome can also be visualized on the browser. Various modules for the functional annotations of the host and pathogen proteins such as subcellular localization, functional domains, gene ontology annotations, pathogen orthologs and effector proteins have been implemented. The host proteins that serve as transcription factors, along with the respective Kyoto Encyclopedia of Genes and Genomes (KEGG) pathways are also available, which further enhance the understanding of the disease infection mechanisms and the defense responses of the host. The database is also linked with several other databases such as InterPro, KEGG pathways, Ensembl and National Center for Biotechnology Information (NCBI). TRustDB has a user-friendly web interface, which can be accessed through .

**Database URL** http://bioinfo.usu.edu/trustdb/

## Introduction

In the past decade, the impact of infectious diseases in plants has been a primary concern due to the increased incidence of highly virulent pathogens in major crops of the world. Over the course of evolution, various new pathogen strains are emerging that cause highly infectious plant diseases, thus causing huge economic and agricultural losses worldwide (1). Wheat (*Triticum aestivum* L.) is considered as one of the most important staple food grains and is a source of essential nutrients such as carbohydrates, vitamins, proteins, minerals, phytochemicals and others. The future global production of wheat needs to be substantially escalated due to the increased consumption and changing dietary habits of people (2, 3), which can be accomplished through sustainable agriculture strategies. Apart from the high demand for the crop, wheat production is also affected by pests and diseases that accounts for about 21.5% of the crop yield losses (4). The impact of the disease varies annually, depending on the incident rate and pathogenesis of the pathogen, resulting in reduced grain quality and quantity (5).

Biotrophic rust fungi are considered a major constraint in the wheat production and have many strains or races that cause peculiar infections in wheat (6). Recently, wheat stem (or black) rust has been of great significance to researchers, owing to its evolved pathogenic mechanism that overcomes the race-specific host resistance genes. The disease, caused by *Puccinia graminis* f. sp. *tritici* Ericks and Henn (*Pgt*), has been detected in many countries around the globe and is associated with high yield losses, reduced grain size and water loss in the crop (7). Over time, *Pgt* evolved into many strains, of which the African strain ‘Ug99’ (race TTKSK) and the Australian strain ‘21-0’ are of major focus (8, 9). Stem rust infection initiates with the germination of urediniospores, followed by the differentiation of mother cells into haustoria that intakes the nutrients from the host cell (10). The effective fungicides to control the disease are available but the unique mechanism of pathogens develops resistance against the chemicals. Also, the usage of chemicals increases the production cost and is harmful to the environment (11, 12).

The pathogens subvert the host cellular mechanisms during the infection process, while the plant cells involve gene-for-gene resistance that activates various downstream immune signaling events such as salicylic acid pathway, reactive oxygen species (ROS) production, mitogen-activated protein kinase (MAPK) signaling and others, which are interconnected with the transcription factors (TFs; 13, 14). Furthermore, the TFs are also considered as positive regulators during biotic stresses (15). Thus, the protein–protein interactions (PPIs) play an important role in both pathogen and host species to cause the infection and initiate defense responses against the disease, respectively (16). The complex network of PPIs between the host and pathogen proteins can be analyzed in an efficient manner using the computational systems biology approach, which reveals the biological information at multiple levels (17, 18).

Various studies in the past have mapped stem-rust-resistance quantitative trait locus (QTLs)/genes in wheat using genome-wide approaches (19). Numerous host–pathogen PPI data sources are available for different pathosystems but no open resources are available for the retrieval of the biological information of the proteins involved in PPIs for stem rust disease. In line with this, we developed a user-friendly, open-source web server ‘TRustDB’ that implements interolog-based PPI prediction and provides functional annotations of the host and pathogen proteins involved in the interactions during stem rust infection in wheat. The database provides a comparative study of the proteins of two Puccinia species responsible for causing stem rust disease in wheat. The broad goal of our study includes not only the protein annotation of the predicted PPIs but also providing the research community with a robust framework that enhances their understanding of the biology underlying the disease infection mechanism. TRustDB is freely available for public use at http://bioinfo.usu.edu/trustdb/.

## Database overview

TRustDB is an extensive resource, composed of the functional annotations of the proteins involved in stem rust disease in wheat. Three diverse tools: ‘interactomics’ tool to predict the host–pathogen interactions, ‘advanced search’ module for the keyword-specific search of the proteins and ‘BLAST search’ are implemented on the database. The tools provide enhanced visualizations for the retrieval of relevant protein information. Furthermore, various functional annotations of the host and pathogen protein in the form of features have been implemented on the database that gives protein-specific information obtained from different sources. The database provides a user-friendly environment for novice users to obtain the required information for the host and pathogen protein annotations. Additionally, the ‘User Guide’ page implemented on the database helps the user to browse the database efficiently.

## Dataset compilation

For the construction of the database, the proteome of *T. aestivum* was obtained from Ensembl Plants (http://ftp.ebi.ac.uk/ensemblgenomes/pub/release-51/plants/fasta/triticum_aestivum/pep/). On the other hand, the proteomes of *Pgt* Ug99 and *Pgt* 21-0 were obtained from Ensembl Fungi (http://ftp.ebi.ac.uk/ensemblgenomes/pub/release-51/fungi/fasta/puccinia_graminisug99/pep/) and NCBI (https://www.ncbi.nlm.nih.gov/assembly/GCA_008522505.1), respectively. All the proteomes were individually analyzed with Cluster Database at High Identity with Tolerance (CD-HIT) at 100% identity to remove redundancy, resulting in 104 701, 22 524 and 35 376 proteins of *T. aestivum*, *Pgt* Ug99 and *Pgt* 21-0, respectively. The information of these proteomes has also been made available in ‘Datasets’ section of the database, which allows the user to download the proteomes from their respective resources. Different tools were used for the prediction of the functional annotations of the host and pathogen proteins, which are described in the respective features section.

## Database architecture and web interface

**Figure 1.**
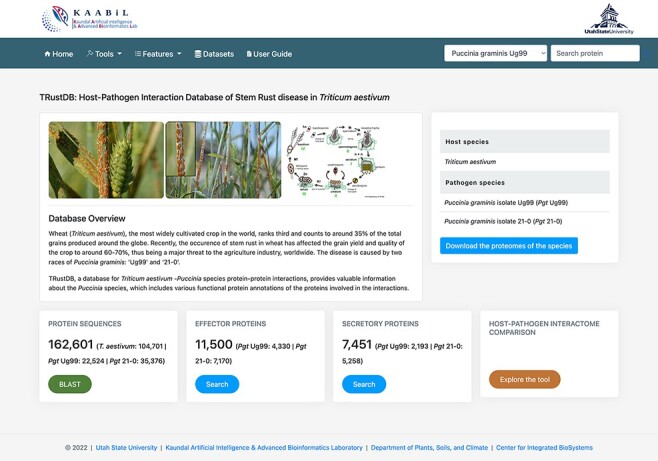
The ‘Homepage’ of TRustDB database server.

TRustDB is hosted on a CentOS Linux system in a High-Performance Computing cluster facility using a virtual machine. The web server runs using Apache HTTP server v2.4.41 and is designed using multiple scripting languages: MySQL as the data storage engine; PHP scripts for querying the MySQL tables and HTML/CSS/Bootstrap 4/JavaScript stack for the enhanced frontend. Figure 1 represents the homepage of TRustDB.

## Prediction tools implemented on TRustDB

### Interactomics: predict host–pathogen interactions

**Figure 2.**
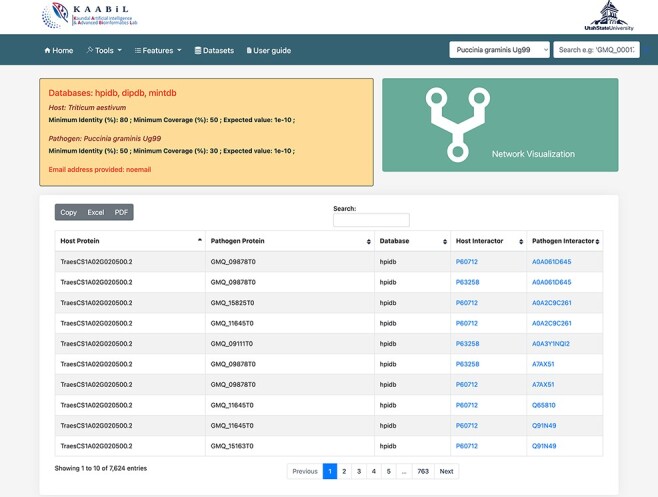
The ‘Table’ view of resulting host–pathogen interactions. The interologs of the corresponding host and pathogen query proteins are also provided. Results can be downloaded in an Excel or PDF format or can be copied to the clipboard.

**Figure 3.**
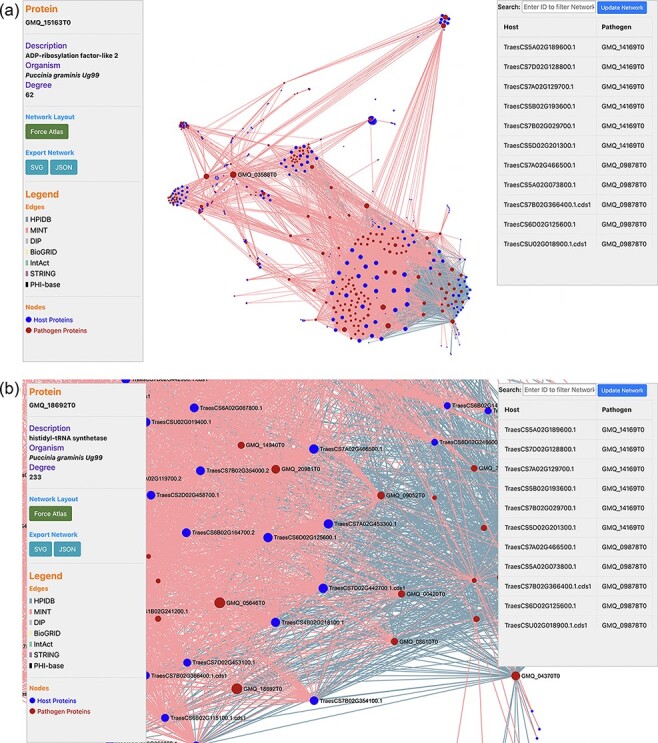
(a) Network visualization of the predicted interactome from the ‘Interactomics’ tool; (b) A zoomed-in version of the network to view proteins of interest. Users can click (or hover) on any node to view the description of a particular host/pathogen protein in the predicted network.

PPIs are the most crucial component in causing the disease in plants. Hence, the prediction of the potential PPIs enhances the understanding of the disease infection mechanism and the defense response generated by the host cells (20). TRustDB provides a comprehensive tool, known as ‘Interactomics’, that predicts the interolog-based interactions between host and pathogen proteins. Interolog approach relies on the sequence homology of the proteins between two species. At the backend, the host and pathogen proteomes were Basic Local Alignment Search Tool (BLAST) searched against the seven standard PPI databases (HPIDB, MINT, DIP, STRING, BioGRID, IntAct and PHI-base), and the resulting alignments were stored. For PPI prediction, the user can select the known database(s) of their choice, input the alignment filtering options (*e*-value, % identity and % coverage) and enter the email address to receive the notification about the job completion. Following this, the stored BLAST alignments are used to predict the interactions using R script and SQL functions, locally. The resulting interactions can be downloaded in Excel or PDF format or can also be copied to the clipboard (Figure 2). Furthermore, the predicted interactions can be visualized on the web interface, which is implemented using the JavaScript-based library, SigmaJS (https://www.sigmajs.org/). This library is very flexible and can handle a large number of interactions very efficiently. The resulting network (Figure 3a) is represented by different colors that represent different known PPI database(s) selected initially and can also be downloaded in SVG and JSON format, with JSON being the highly acceptable format by most of the network analyzers for further enhancement of the network. Users can zoom-in on the network visualization to view any proteins of interest (Figure 3b); the description of a particular host/pathogen protein in the predicted network can be obtained by hovering over the protein of interest. The results from the interactomics tool are also stored on a backup server for 30 days.

### Advanced search module

**Figure 4.**
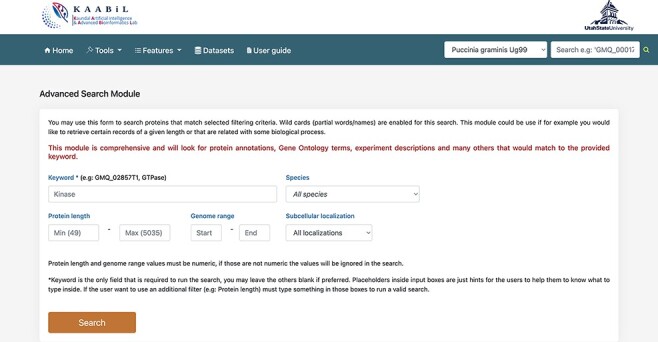
A snapshot of the ‘Advanced search’ module of TRustDB; users can search the database using specific keywords, protein IDs and/or other genomic information of the proteins.

The ‘advanced search’ interface of TRustDB provides comprehensive functionality to search the database using specific keywords and/or genomic information of the proteins (Figure 4). This module extracts the related proteins by searching multiple SQL tables in accordance with the user query. The users can also use the ‘basic’ search available under the ‘Features’ menu on the database. The user can enter biological keyword of choice to obtain all the proteins related to the query. Additionally, the user can also give a particular value for the protein length or genome range in the query, otherwise the program filters out all the proteins for the specific keyword in the results. A specific subcellular localization can also be selected from the dropdown menu, which enables the user to search the database for the proteins in the particular subcellular localization. The search outputs the proteins for the queried keyword along with information such as gene coordinates, protein length, description of the protein and gene ID associated with the proteins. Furthermore, on selecting any protein ID, the user will be taken to a new page that displays additional information of the protein such as NCBI reference ID, locus tag, InterPro annotations and functional domains of the particular protein, if available. From this page, the user can also obtain the amino acid sequence of the protein. The external database IDs such as NCBI, InterPro and others are linked to the respective databases. The interconnection of various features such as subcellular localization, InterPro annotations, functional domain mappings and genomic information makes the ‘advanced search’ an ideal, time-efficient tool for the users.

### BLAST search

**Figure 5.**
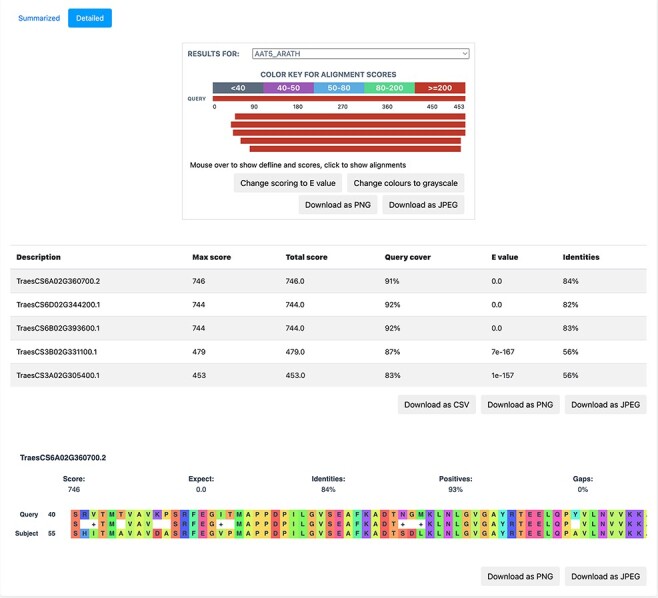
A detailed view of the BLAST result alignments.

BLAST is based on sequence homology and finds the similarity between two sequences. We implemented BLAST on the database to provide a functionality, whereby the users can query the nucleotide/protein of interest against the available proteomes on TRustDB. The user can submit either nucleotide or protein sequences, which are automatically detected by the program, followed by performing BLASTp or BLASTx alignment, accordingly. A choice of specific weight matrix in BLAST can also be made from the available options. The results of BLAST search are available to be downloaded in PDF or Excel file formats, with an option to download the alignments as a text file. Additionally, a detailed version (Figure 5) of the BLAST alignments is available that includes the alignments in graphical format for enhanced visualization, which is implemented using the JavaScript-based library, BlasterJS (21). These grayscale/colored alignments can be downloaded in JPEG or PNG format.

## TRustDB features: Annotations of host and pathogen proteins

The primary goal of TRustDB is to provide the researchers with the functional annotations of the host and pathogen proteins for a better understanding of the protein function during disease infection mechanisms. The proteins were analyzed using various prediction tools to obtain the annotations such as subcellular localization, gene ontology annotations, functional domain mappings, Puccinia orthologs, effector proteins, secretory proteins and host TFs. These annotations are implemented individually in the ‘Features’ section of the database. Apart from the interactomics tool, the pre-calculated host–pathogen PPIs between *T. aestivum* and Puccinia species are available in the ‘Host-pathogen interactions’ module under the ‘Features’ menu, based on default BLAST alignment filters (*e*-value 1e−04, sequence identity 30% and sequence coverage 40%). These pre-calculated interactions were predicted using our in-house python scripts, and the BLAST parameters combination was selected based on the highest number of effector and secretory proteins obtained in the predicted interactions. More details about the schema followed for pre-calculated interactions can be found under the ‘information icon’ of the ‘Host-pathogen interactions’ module of TRustDB. Similarly, most of the feature modules contain the ‘information icon’ that gives brief information about the data available on that particular page. A few of the functional annotations such as subcellular localization, gene ontology (GO) term annotations and functional domain mappings are implemented for both host and pathogen proteins in a separate sub-section for host and pathogen under the features. To obtain the subcellular localization of the host proteins, the proteins were analyzed using the SVM-based tool Plant-mSubP (22), while the pathogen proteins were analyzed in DeepLoc 1.0 (23). Furthermore, the standalone version of InterProScan (24) was used to obtain the GO term annotations and functional domain mappings of the host and pathogen proteins. These modules provide the user with the conserved domains and GO terms associated with the specific protein. The InterPro accessions and domain names have been provided, along with their respective descriptions.

There is a wide range of *P. graminis* strains, which evolved from the African race ‘TTKSK’ (Ug99, named after stem rust infection in Uganda in 1999) (25). Based on this, we predicted the orthologs of *Pgt* Ug99 and *Pgt* 21-0 using the tool ‘OrthoFinder’ (26). This information is available on the database and contains a unique ‘Ortho Group’, corresponding to the *Pgt* Ug99 and *Pgt* 21-0 proteins that are orthologs of each other. The user can also avail the protein sequence of the orthologs by clicking the identifiers under the ‘Ortho Group’ tab. The pathogens secrete effector and secretory proteins into the host cells for proliferation and successful infection, thus subverting the host cell physiology and suppressing the defense responses (27, 28). Thus, we implemented the modules for the pathogen proteins that serve as effector and secretory proteins separately on the database. The effector and secretory proteins were predicted using the tools EffectorP 2.0 (https://effectorp.csiro.au/) (29) and SignalP 5.0 (https://services.healthtech.dtu.dk/service.php?SignalP-5.0) (30), respectively. The information about the pathogen protein description and length is also available in these modules. Furthermore, the TFs in the host are known to play a critical role in defense mechanisms by regulating various biological processes and genes involved in the plant immune responses during various biotic and abiotic stresses (31). The TFs for *T. aestivum* proteins were predicted using PlantTFDB v4.0 (http://planttfdb.gao-lab.org/) (32), which is an extensive resource of TFs. Each of the TFs is externally linked to its respective transcription factor family, which gives more information about the TF family. In this module, we have also provided the users with the information on host Kyoto Encyclopedia of Genes and Genomes (KEGG) pathways and their respective KEGG descriptions, which are linked to the KEGG pathway database.

## Use case and database validation

**Figure 6.**
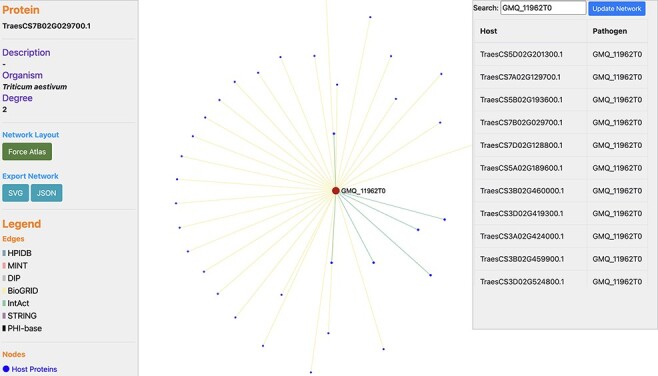
Network visualization of an Ug99 effector protein, GMQ_11962T0, interacting with 34 *T. aestivum* proteins as predicted by TRustDB (Supplementary Material, Sheet 4). GMQ_11962T0 has been shown to be involved in hydrolase activity (GO:0016817) and spliceosome (ko03040), which plays a role in the colonization and pathogenicity of the fungal pathogens.

In wheat, the disease resistance to stem rust is based on the ‘gene-for-gene’ resistance concept (33). In the past, various QTLs and resistance (R) genes (Sr5, Sr13, Sr22, Sr33, Sr45 and others) against stem rust disease have been identified on wheat chromosomes 1B, 2B, 3B, 5A, 5D, 6A, 6D, 7A and 7B (34, 35). We identified the proteins related to these chromosomes, which resulted in 35 667 wheat proteins, interacting with 1717 pathogen proteins (Supplementary Material, Sheet 1 and Sheet 2, respectively). Among these proteins, 288 host proteins were identified to serve as TFs and 17 pathogen proteins were found to be effectors (Supplementary Material, Sheet 3). For example, Ug99 effector protein ‘GMQ_11962T0’ interacts with 34 *T. aestivum* proteins (Supplementary Material, Sheet 4), as can be visualized in a network (Figure 6). For experimental/literature validation of the PPI pairs, we randomly selected the PPI pair ‘TraesCS1B02G424700.1 - GMQ_11962T0’ from the predicted interactome. The functional annotations of the host protein ‘TraesCS1B02G424700.1’ revealed that the protein is involved in plant-defense-related biological processes such as plant hormone signal transduction pathway (ko04075) and cellular response to auxin stimulus (GO:0071365). This protein was also found to belong to the TF family ‘ARF’ (i.e. Auxin Response Factor) and localized in the nucleus of cell. Researchers in the past have reported the role of signaling pathways such as abscisic acid pathway and jasmonic acid (JA) pathway in the regulation of stomatal closure during biotic/abiotic stress conditions (36). Another research demonstrated that JA and ethylene were required for the activation of the defensin gene (PDF1.2) that was found to be induced on fungal attack (37). These reports show the significant role of the plant hormones in plant defense against the pathogen attack. On the other side, the interacting pathogen protein ‘GMQ_11962T0’ was found to be involved in hydrolase activity (GO:0016817) and spliceosome (ko03040), which plays a role in the colonization and pathogenicity of the fungal pathogens. Several glycoside hydrolase (GH) families have been identified that serve as effectors and play a critical role in the pathogenicity of fungi. These are responsible for initiating various diseases in plants, for example GH3 is responsible for leaf spot disease and GH10 is responsible for vascular disease in tomato. A GH12 protein (PsXEG1) resulted in triggering the cell death in soybean (38). Furthermore, a study on *Sclerotinia sclerotiorum* indicated that the alternative splicing events occur during fungal colonization and are responsible for functional diversity of the proteins (39). To further verify the database, we also queried the host and pathogen protein in the above-mentioned interaction pair on different modules available in the ‘Features’ menu in the database. The annotations of the proteins searched on TRustDB were found to be similar as those obtained from the respective annotation resources for GO/KEGG, thus validating the annotations available in the database.

Additionally, we also deciphered some other functional differences between the two pathogen strains used in the study. For example, we found that the *Pgt* Ug99 strain was involved in monoterpenoid biosynthesis pathway, snoRNA binding (GO:0030515) and GTPase complex (GO:1 905 360). Meanwhile, the *Pgt* 21-0 strain was involved in plant hormone signal transduction pathway, carotenoid biosynthesis, SUMO ligase complex (GO:0106068) and cell wall modifications (GO:0042545). These significantly enriched GO terms and KEGG pathways regulate the fungal growth and development during the infection, as reported earlier (40–43).

## Conclusion and future directions

The data available on TRustDB are a beneficial host–pathogen PPI resource for molecular biologists for analyzing and understanding the infection mechanisms of stem rust disease in *T. aestivum* and for the breeders to develop disease-resistant crop lines, thus enhancing the yield and quality of the crop. In the near future, the database is expected to be augmented by incorporating other important rust diseases of wheat.

## Supplementary Material

baac068_Supp

## Data Availability

The database is freely available for public use at http://bioinfo.usu.edu/trustdb/. The datasets explored in this study to develop the computational models are also available on this website. Supplementary data/results are available in one Excel file containing four different sheets.
